# The case space model within the OSCE framework: more clarity for the SP, examiners and students 

**DOI:** 10.3205/zma001494

**Published:** 2021-09-15

**Authors:** Ulrich Pleines Dantas Seixas, Thomas Speier, Lilian Künzler

**Affiliations:** 1 Universität Basel, Medizinisches Studiendekanat, Basel, Switzerland

**Keywords:** OSCE, simulated patients, examiner, candidate, understanding of one's role, case space model, authenticity, believability, certainty

## Abstract

**Objective:** Simulated patients play a significant role in OSCE (Objective Standardized Clinical Examination) settings. Does the understanding of one's own role or task lead to more clarity for all protagonists involved in an OSCE?

**Methodology: **Based on the range of professional experiences and qualifications of the training team it was possible to bundle medical, acting and didactic knowledge.

It was against this background that the individual understanding of one’s role was examined from its respective points of view.

**Result: **As a result, the authors have developed the so-called Case Space Model, which encourages critical exchange and discussion. It is a visualization of the relationships between the simulated patients, examiners and candidates. During the course of the model’s development a preference for the term believability instead of authenticity became clear.

**Conclusion: **The Case Space Model can help to clarify the positions and provide certainty in the respective roles.

## Zusammenfassung

**Zielsetzung: **Simulationspersonen spielen bei OSCE-Prüfungen (Objectiv Standardized Clinical Examination) eine tragende Rolle. Führt das Verständnis der eigenen Rolle oder Aufgabe der an einem OSCE beteiligten ProtagonistInnen zu mehr Klarheit?

**Methodik: **Aus der Mischung der beruflichen Herkunft und Qualifikationen des TrainerInnenteams war es möglich, medizinisches, schauspielerisches und didaktisches Wissen zu bündeln. Ausgehend von diesem Hintergrund wurden die einzelnen Rollenverständnisse aus ihrer jeweiligen Sicht betrachtet.

**Ergebnis:** Das Ergebnis ist das von den AutorInnen so genannte Rollenraum-Modell, welches zum Austausch und diskutieren anregen soll. Es macht die Beziehungen zwischen Simulationspersonen, PrüferInnen und KandidatInnen sichtbar. Im Verlauf der Entwicklung des Modells zeigte sich die Präferenz des Begriffs Glaubhaftigkeit anstelle von Authentizität.

**Schlussfolgerung:** Das Rollenraum-Modell hilft dabei, die Positionen zu klären und gibt Sicherheit in der jeweiligen Rolle.

## Introduction

Within the framework of experiences with simulated patients (hereinafter: SP) the wish arose amongst us trainers to gain more clarity on the positions and roles of the individuals actively participating in an OSCE, and in what relationship they stand to one another. The result of our observations and reflections, which are derived from practical experience, is presented here as the Case Space Model

## Setting the stage

During work with the SPs two main problems became apparent. On the one hand we observed that in some instances of an OSCE the comparability of a role’s portrayal was only marginal, on the other that the role playing appeared more like a character imitation than a believable interpretation. 

The first, intuitive [1] step towards change was to consciously remove the SP from our center of awareness ([2] p.8f) and instead insert the candidates in that place, as they are the main party involved in an OSCE. 

The next step was then to add a well-trained supporting cast – the SPs – into the thus prepared stage [3], so that the main protagonists – the candidates – could have the opportunity to prove their ability under the optimal circumstances available to them. The examiners were given the best seats in the audience from where they could become involved when asked to from the stage, or actively intervene when necessary (information space).

## Contact between SP and candidate

**Figure 1:**
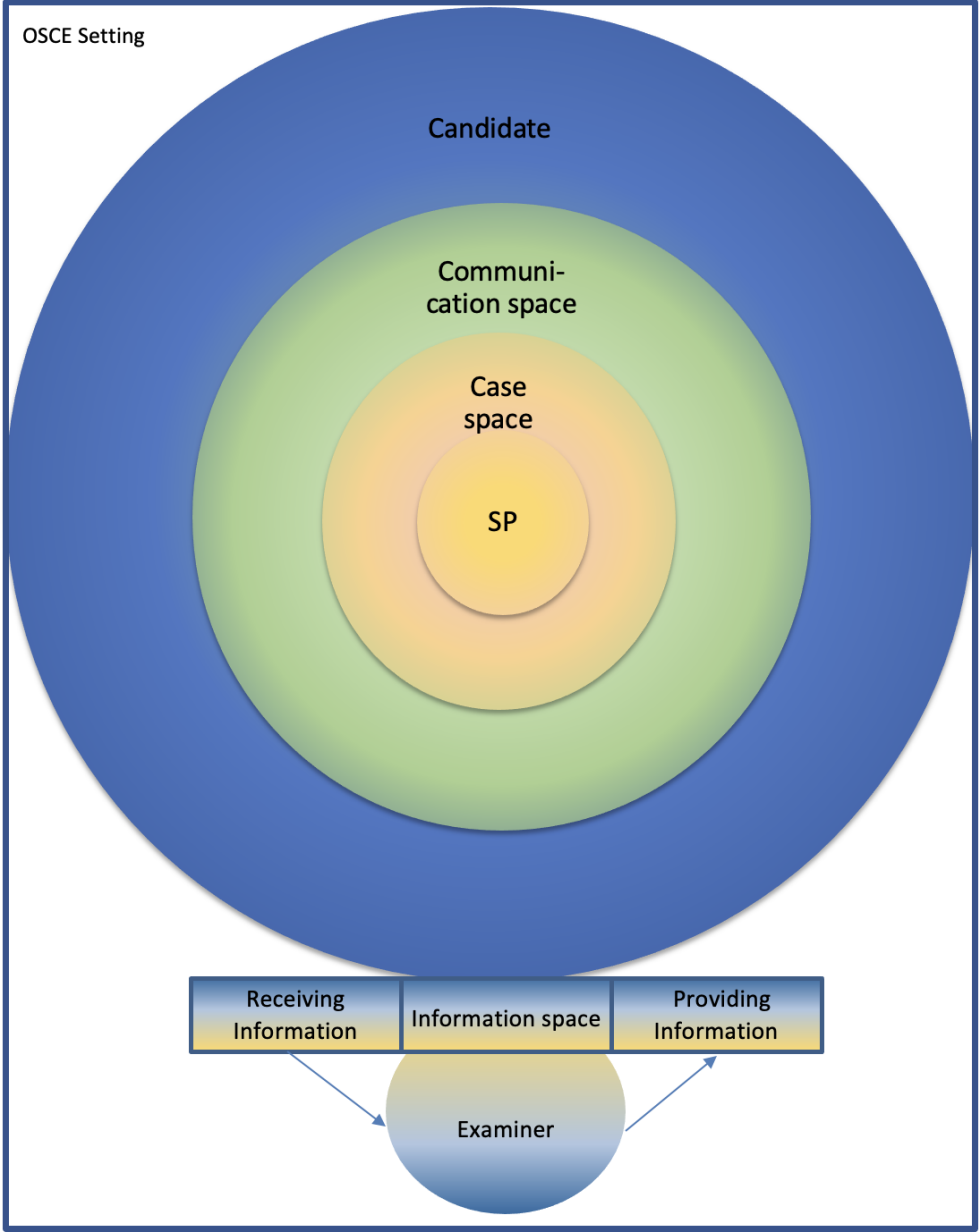
OSCE structures

In accordance with their scripted role, the SPs move within an area that we have designated as the case space. The candidates move within their exam reality and at the same time in a role-play situation. Contact between the case space and the candidates is made through the communication space. As long as the candidates communicate – or are in contact – with the SP, the latter will always have the opportunity to behave in conformity with their role or examination scenario. The communication space serves as a link between the acted role and the candidates. Structures presented by the respective OSCEs make up the external boundaries of the model (marked as OSCE setting in figure 1 (Fig. 1)). 

The SPs may move freely about within the constraints set by the standardized role. The limits are defined by information provided in the role's script. During training, the SPs are specifically led to these boundaries through the application of techniques acquired from acting, so that they can get to know these limits.

## Contact between candidate and examiner

The examiners in an OSCE are expected to restrain their interventions as much as possible. This is defined by the OSCE system, as it is a standardized examination procedure. Every contact with the candidates risks an oral intervention and therefore a subjectivization. The Case Space Model places the examiners on the outside. Depending on the case, they have the possibility or duty to provide the candidates with information (for example: examination reports, lab results) or to receive communications from them (suspected diagnosis, further examination procedures). 

## Contact between the SP and the examiners

The SP and the examiners should influence one another as little as possible. As a matter of illustration, they have no point of contact in the case space model. But since they do spend a considerable amount of time together in the examination room, interaction will naturally occur. This interaction must not be allowed to affect the result of the examination. In a formative setting, e.g. the use of SPs in teaching and not in an examination, the latter point only has a limited validity. But even here all assessors must reach their results independently and from their own perspective. 

## Look carefully

A superficial glance may lead one to think that the SP takes center stage in the model. But this is deceptive, as a closer examination will quickly make it clear that the candidates occupy most of the space. The candidates move in the awareness of the examination situation. The OSCE system implies that the candidates want to convince the examiners of their capabilities. With the help of the SP they are delivering a performance for the examiners. 

## Appearance and reality?

We changed the aspiration of our training in such a way, that it is not the patient's role to be authentic in the sense of realness, but instead that it should convey believability (=plausibility; something that deserves to be believed for a reason) ([4] p.101]. The only thing authentic about an OSCE situation is that it is an examination setting, in which a well-educated person playing the role of a doctor encounters a well-trained individual who believably portrays an impaired (ill) person. 

During training with clinicians it became apparent, that they often recognized several “types” of patients with their “image” as possible and acceptable, for all intents and purposes they were authentic and known from their everyday experience in the clinics. This led to confusion and discussions among the SPs, when they assumed they were portraying a certain authentic patient. This contradiction can be solved in terms of believability, when different people with their expressions and senses of their own individuality use the same symptoms and behavior regarding communication to convincingly represent an ill person. The SP as a role-playing figure tells their story in such a believable manner, that everyone involved is under the impression that: yes, this is how it could have played out. 

## Conclusion

The Case Space Model illustrates in fundamental systematics the positions of the individuals involved in an OSCE. The graphic structuring of the OSCE setting allocates the protagonists their respective place. By recognizing and defining one’s own role, embedded within the particular function, the participants obtain clarity in regard to their task, and the understanding of that role becomes clear. 

## Competing interests

The authors declare that they have no competing interests.
